# Towards Thin Calcium Metal Anodes—An Essential Component for High-Energy-Density Calcium Batteries

**DOI:** 10.3390/nano15060454

**Published:** 2025-03-17

**Authors:** Christoph Kiesl, Reinhard Böck, Holger Kaßner, Joachim Häcker, Marco Kögel, Timo Sörgel, Şeniz Sörgel

**Affiliations:** 1 Department of Electrochemical Energy Systems, Research Institute (fem), Katharinenstraße 13-17, 73525 Schwaebisch Gmuend, Germany; 2 Center for Electrochemical Surface Technology (ZEO), Aalen University of Applied Sciences, Beethovenstraße 1, 73430 Aalen, Germany; 3 Institute of Engineering Thermodynamics, German Aerospace Center (DLR), Pfaffenwaldring 38-40, 70569 Stuttgart, Germany; 4 Natural and Medical Sciences Institute, University of Tuebingen (NMI), Markwiesenstraße 55, 72770 Reutlingen, Germany

**Keywords:** calcium, multivalent metal battery, metal anode, electroplating, electrodeposition, electrocrystallization

## Abstract

Metal anodes, such as those based on Ca, Mg, Na and Li, are considered to be one of the keys to the further development of high-energy-density rechargeable batteries. The thickness of these metal anodes directly affects the energy density of the battery. However, the fabrication of thin anodes poses technical challenges which often result in using excessively thick metal anodes in batteries. Here we present, for the first time, a study on the development of a thin Ca battery anode fabricated by electrodeposition. The battery anode with a thickness of approximately 10 µm corresponds to a charge density of 4.0 mAh cm^−2^. This study systematically investigates the electrodeposition behavior of Ca using a 1.0 M Ca(BH_4_)_2_ in THF as the electrolyte. A systematic evaluation of electrodeposition parameters—including substrate pretreatment, current density, hydrodynamics and charge density by area—is conducted. Scanning electron microscopy (SEM) and complementary image analysis provide detailed insights into these parameters. Electrodeposition offers a promising route to achieve a defined battery cell balance with minimal excess of metal at the anode. This will improve overall battery performance and efficiency. The findings contribute to the advancement of fundamental aspects of rechargeable batteries, particularly Ca-based batteries.

## 1. Introduction

The starting point of a great success story goes back to 1985, when Akira Yoshino invented the first safe and lightweight rechargeable battery based on intercalation type active materials, the Lithium Ion Battery (LIB) [1]. At present, the LIB offers a gravimetric energy density over 270 Wh·kg^−1^ [2]. However, it is expected that the LIB will not be able to meet the growing demand for future energy storage devices. Technical challenges associated with metallic Li anodes, as well as the demand for sustainable materials, are the main drivers for reducing Li dependency and moving beyond Li-based batteries [3,4]. The development of post-LIBs, which have the potential to offer superior energy density, reduced environmental impact and lower cost with higher safety aspects, is therefore of considerable interest [5,6]. The concept of using metal anodes emerges as one of the leading contenders in this context, with Mg, Al and Ca being among the top ten most abundant elements in the Earth’s crust, which also has a positive impact on the cost situation [6,7,8,9]. Notably, Ca represents a very attractive candidate, with a standard reduction potential in aqueous solutions of −2.87 V vs. SHE (0.17 V more positive than Li) [10] and a high volumetric and gravimetric capacity of 2073 Ah L^−1^ and 1337 Ah kg^−1^, respectively [11]. Early studies identified the challenge of achieving reversible Ca deposition. This was due to the passivation layers formed, mainly composed of CaCO_3_ and Ca(OH)_2_, related to side reactions inhibiting subsequent plating [12]. Recent advances have demonstrated reversible Ca plating and stripping using specialized electrolytes, although side reactions and temperature dependencies remain significant obstacles [13]. In contrast to the ongoing development of electrolytes, the challenges associated with the production of a metallic Ca anode are rarely addressed in the literature. Most studies apply thick Ca foils (>250 µm) or pressed Ca powder discs (>800 µm) with a large Ca excess. For the transition from laboratory scale to practical application, the implementation of a thin anode will be important for the achievement of a well-balanced battery cell with a high-energy density [14,15]. The high reactivity of Ca with atmospheric constituents, and the stiffness of Ca, which is four times greater than that of Li (Young’s modulus Li: 4.9 GPa; Ca: 20 GPa), are major challenges and may preclude the processing of a thin Ca anode using existing anode manufacturing processes [16]. As a result, commercially available Ca foils are currently limited to dimensions on a laboratory scale (small area and a minimum thickness of 100 µm).

Herein, we report for the first time the fabrication of a Ca anode by electrodeposition which offers the opportunity to balance its thickness regarding the capacity of the active material at the cathode. It is shown by tuning the electrodeposition process, a compact Ca anode with a minimum thickness of 10 µm can be obtained. Furthermore, the use of electrodeposited battery components offers several advantages, including resource and cost optimization, increased energy density and enhanced electrical conductivity, as demonstrated in some previous research [17,18,19,20]. Thus, reducing the amount of metal by employing a thin metal anode will contribute to higher safety. In addition, the morphology and the SEI of the electrodeposited layer can typically be modified, which may lower the overpotential contributions of the anode and provide the opportunity to improve the energy efficiency. The electrodeposition process is a well-established technology which is widely used in various industries. The process itself can be described by the application of an overpotential (the excess voltage required for a net reaction to take place), which induces a current through a circuit containing an electrolyte with two electrodes. The formation of the metallic layer of interest begins to form at the negative electrode (cathode). Simultaneously, an oxidation process proceeds at the counter electrode (anode). The initial phase of the deposition at the cathode, known as electrocrystallization, results in the nucleation and growth of crystals. While compact, continuous layers typically form during electrodeposition, the morphology of the layer can be influenced by several factors. These include the electrolyte composition and additives [21,22]. The overpotential instead primarily governs the balance between nucleation and growth rates [23]. At low overpotential, slower deposition rates lead to fewer nuclei, allowing the growth of larger crystals. In contrast, higher overpotentials shift the balance, significantly increasing the nucleation rate and resulting in numerous small nuclei. At moderate overpotentials, crystal growth becomes dominated by charge transfer mechanisms and faceted crystals often develop [24]. However, once the overpotential reaches a level where ion diffusion becomes the limiting factor, the morphology becomes prone to the formation of nodules and dendrites (diffusion-limited aggregation) [25,26]. The mechanism of crystal growth under real (non-ideal) electrochemical conditions in aqueous electrolytes can be traced back to Fischer [27], who was the first to draw an integrated picture of the interrelationship between the classical electroplating parameters (current density, convection and temperature), the electrolyte composition (metal ion concentration, inhibitor type/strength and concentration) and the so-called growth forms, a term introduced by Fischer to describe the appearance of the deposits, either to the naked eye or as seen through a light microscope (cross-sectional view).

This research article is motivated by the necessity of a reliable and cost-effective manufacturing process forming a thin Ca metal anode and delves into the distinctive advantages presented by the electroplating process. We approach the interpretation of our new findings by applying general concepts of electroplating from aqueous electrolytes to create a fundamental basis of understanding, mainly focusing on the conventional electroplating parameters (pretreatment, current density, hydrodynamics and charge density per area). Building on this framework, we systematically investigate the electrodeposition behavior of Ca using 1.0 M Ca(BH_4_)_2_ in THF as the electrolyte. Compared to other Ca-containing electrolytes, this system exhibits high coulombic efficiency at room temperature, showing potential for creating suitable electrodeposited Ca anodes. In contrast, inferior performance in other systems is often attributed to passivation layer formation, as described above [12,13,28]. Imaging techniques such as scanning electron microscopy (SEM) and confocal microscopy are used to visualize and analyze the morphology and topography of the Ca deposit.

## 2. Materials and Methods

**Figure 1:**
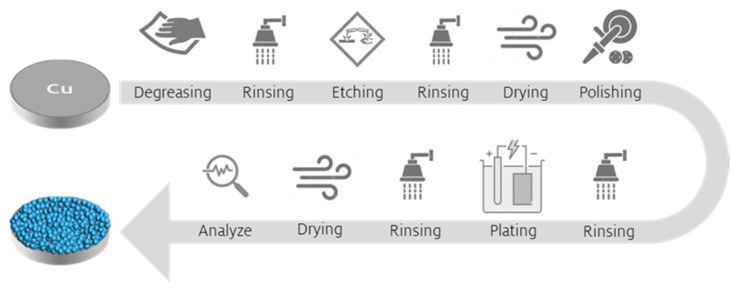
Schematic illustration of the fabrication process for electrodeposited Ca anodes.

Every stage, from handling air-sensitive materials to preparing the electrolyte, assembling the plating cell and conducting experiments, was carried out within a controlled, dry argon atmosphere inside a Glovebox (99.999% Ar, p (O_2_) < 0.5 ppm, p (H_2_O) < 0.5 ppm). The additive free Ca electroplating electrolyte was prepared from anhydrous tetrahydrofuran (THF, purity > 99.9%, Sigma Aldrich, St. Louis, MO, USA) and calcium borohydride bis(tetrahydrofuran) (Ca(BH_4_)_2_·2THF, Sigma Aldrich). Powder based Ca discs (diameter 6 mm), prepared by compressing Ca granules (99.5%, 16 mesh, Thermo Fisher Scientific, Waltham, MA, USA), scraped before used, served as counter electrode. Polycrystalline Cu discs (99.9%, thickness 0.25 mm, Wieland, Ulm, Germany) were utilized as the working electrodes. The Cu substrate was pretreated with a degreaser (Slotoclean EL KG, Schlötter, Geislingen an der Steige, Germany) and an etchant (Micro-Etch 910, Umicore, Brussels, Belgium). Unless otherwise mentioned, the substrate underwent a pretreatment process that included cathodic degreasing at a current density of 50 mA cm^−2^ for 300 s and subsequent pickling for 60 s, followed by a mechanical grinding and polishing step using 800 and 2400 sandpaper. Degreasing and pickling were conducted at ambient air, while grinding and polishing took place in a glovebox environment. The detailed steps involved in the manufacturing process of the Ca battery anode are depicted in Figure 1. Ca(BH_4_)_2_·2THF salt (4.28 g) was dissolved into 20 mL THF at a concentration of 1.0 M Ca(BH_4_)_2_. Prior to use, THF was dried over activated molecular sieves (type 4 Å) for several days. The electrochemical deposition experiments were performed using a MPG-2 potentiostat/galvanostat and EC-Lab^®^ software version 11.40 (BioLogic, Seyssinet-Pariset, France). All experiments and measurements were conducted at ambient temperature.

**Figure 2:**
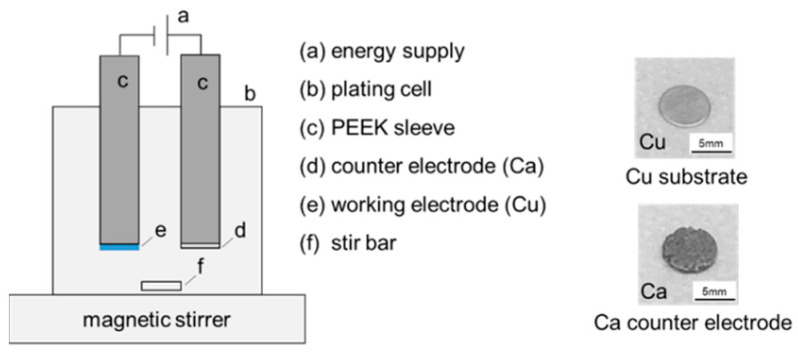
Schematic representation of the plating cell configuration and electrode materials (working electrode: Cu; counter electrode: Ca).

As shown in Figure 2, a semi-gas-tight cylindrical plating cell containing 20 mL of electrolyte and configured with two electrodes was used for the plating experiments. Stainless steel rods covered with polyether ether ketone (PEEK) sleeves were used to connect the substrate disc. A stir bar in combination with an adjustable magnetic stirrer was used to alter the hydrodynamics.

The surface topography of the Ca deposit was characterized using a confocal microscope (Marsurf CM explorer, Mahr, Göttingen, Germany). The surface morphology and chemical composition of the electrodeposits were analyzed using a scanning electron microscope (SEM, Gemini SEM 300, Carl Zeiss Microscopy, Oberkochen, Germany) equipped with energy dispersive X-ray spectroscopy (EDX). To prevent reaction with oxygen and/or moisture, a transfer module (Kammrath and Weiss, Schwerte, Germany) was utilized to safely transport the specimen between the glovebox and SEM. Focused Ion Beam (FIB) investigations were performed in a separate SEM (Auriga 60, Carl Zeiss Microscopy). In a subsequent step, the SEM data were quantitatively evaluated using the open source image processing software ImageJ version 1.54f in combination with the machine learning tool Weka, which was developed by Arganda-Carreras et al. [29]. Through the process of partitioning an SEM image, the tool allows a more thorough quantification of the deposits. The procedure is shown in Figure S1. To complement the assessment of the effect of substrate pretreatment, a contact angle measuring system (OCA 25, Dataphysics, Filderstadt, Germany) was also used.

## 3. Results and Discussion

The first step of our investigation was to study the effects of the electrodeposition parameters. After that, the most suitable experimental condition was selected to manufacture a thin Ca anode. The selected process parameters (Table 1), substrate pretreatment, current density, hydrodynamics (electrolyte stirring) and charge density were chosen based on their fundamental influence on the deposited layer. Substrate pretreatment plays a critical role in nucleation behavior and adhesion, as the surface condition affects the uniformity of the growing layer [30]. By comparing chemical and mechanical pretreatment, we assessed how surface modifications impact nucleation and layer compactness. Current density determines the deposition rate and structure of the deposit [31]. While higher current densities can increase nucleation density and surface coverage, they also risk electrolyte degradation [32]. Hydrodynamics, controlled through electrolyte stirring, influences mass transport and ion availability at the electrode surface. Proper stirring ensures a stable ion flux, reducing concentration gradients and enabling more homogeneous deposition [33]. Lastly, charge density defines the final thickness of the deposited Ca layer. By controlling the total charge passed during deposition, we can directly influence the anode thickness and its balance with the cathode in battery applications. In each series of experiments, one parameter was varied while the others were kept constant to ensure that the results could be accurately attributed to the influence of that single parameter. Detailed information on each experiment is given in the Supplementary Information (Table S1).

**Figure 3:**
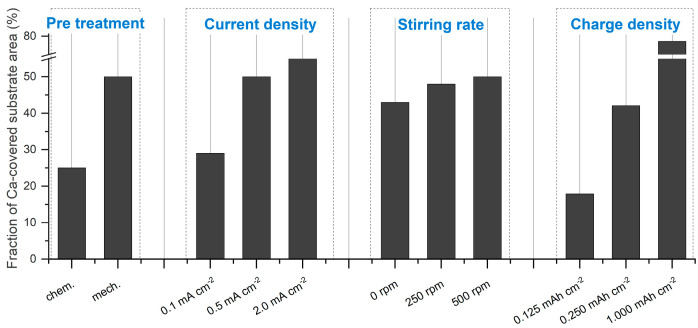
Quantitative data on Ca deposits is presented as fraction of Ca-covered substrate area based on different electrodeposition conditions, including pretreatment, current density, stirring speed and charge density over area.

**Figure 4:**
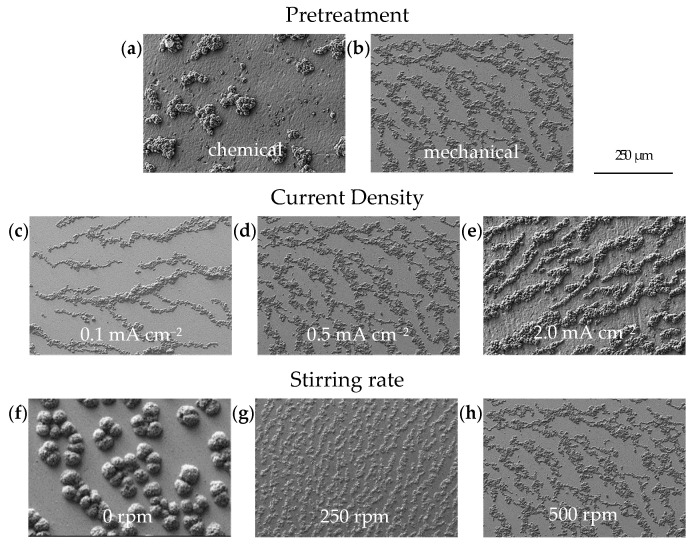

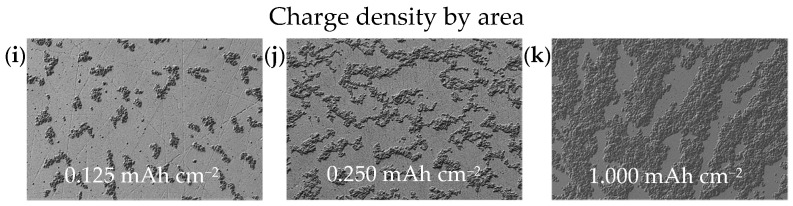
SEM characterization of Ca deposits onto Cu substrates under varying plating conditions: (**a**,**b**) effects of pretreatment, (**c**–**e**) variations in current density, (**f**–**h**) different stirring rates and (**i**–**k**) changes in charge density per area.

**Figure 5:**
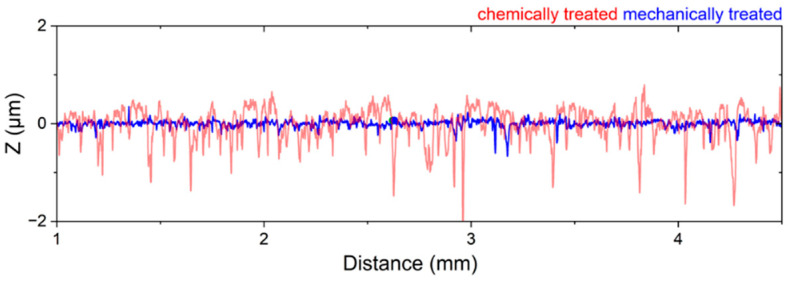
Roughness profile of the chemically and mechanically treated Cu substrates.

**Figure 6:**
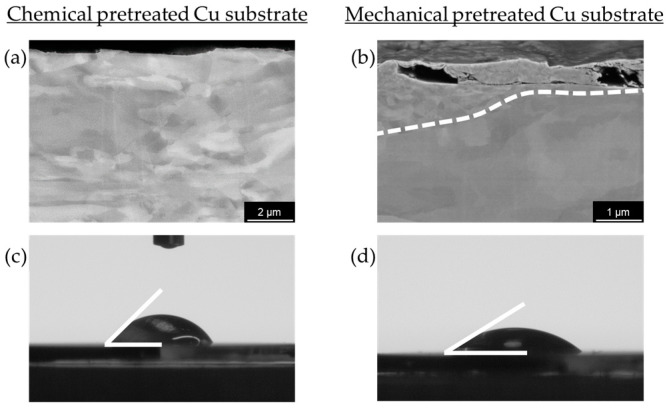
(**a**) Cross-sectional SEM image of Cu substrate—chemically pretreated; (**b**) cross-sectional SEM image of Cu substrate—mechanically pretreated; (**c**) contact angle measurement on Cu substrate—chemically pretreated; (**d**) contact angle measurement on Cu substrate—mechanically pretreated.

The quantitative data of the obtained Ca deposits can be found in Figure 3, while the SEM images of the individual electrodeposition experiments are summarized in Figure 4. Figure 4a shows an SEM image illustrating the typical morphology of Ca deposits on a chemically pretreated Cu substrate under a current density of 0.5 mA cm^−2^ after 30 min (0.25 mAh). The Ca islands exhibit an irregular distribution and vary in size. Similar results, though on a smaller scale, have been reported by Pu et al. [34]. Ta et al. [35] propose that a substrate-dependent chemical reaction step initiates a chemical-electrochemical deposition process, primarily driven by the dehydrogenation of the borohydride anion in the presence of the metal substrate. This process results in a smooth deposit on Au, while a patchier Ca deposit results on Pt. Consistent with this, Duan et al. [36] report that the electrochemical activity, specifically the oxidation of borohydride on Cu, is comparable to that observed on Pt. In contrast, our results show that the deposition on mechanically pretreated substrates changes the arrangement into smaller, coalesced Ca islands that follow the hydrodynamic flow (Figure 4b). The role of substrate pretreatment becomes clear when looking at the plated substrate area values: 25% of the substrate area is plated on chemically pretreated substrates, while a coverage of 50% is obtained on the mechanically pretreated substrate (Figure 3). This significant difference suggests that the pretreatment method directly influences the nucleation process and subsequent deposition behavior. Surface roughness is typically a key parameter influencing nucleation during electrodeposition. Interestingly, despite the expectation that increased roughness enhances nucleation, our mechanical pretreatment substantially reduces the surface roughness compared to chemical pretreatment (Figure 5), yet it leads to a higher nucleation density (Figure 3 and Figure 4b). This finding indicates that the mechanical pretreatment not only smoothens the substrate but also enhances nucleation through alternative mechanisms. Moreover, mechanical surface pretreatment introduces micro-defects, such as dislocations and grain boundary distortions into the substrate, significantly increasing the number of nucleation sites during electrodeposition. Zhou et al. [30] observed that these defects serve as active nucleation centers for metal electrocrystallization, facilitating uniform deposition. Our contact angle measurements (Figure 6c,d) further illustrate the effects of pretreatment on surface wettability, which is closely related to surface energy. Chemically pretreated substrates exhibit higher contact angles, indicating reduced wettability, whereas mechanically treated surfaces display lower contact angles, signifying enhanced wettability. The wetting model proposed by Wenzel [37] predicts that surface structuring affects the apparent contact angle in such a way that the wetting properties specific to the material itself are enhanced in either direction. However, our findings indicate that mechanical pretreatment not only alters surface roughness but also modifies surface characteristics by disrupting the surface layer and creating lattice defects. This disruption potentially reduces the energy barrier for heterogeneous nucleation, creating a more reactive surface for electrodeposition. Our hypothesis is further supported by the SEM cross-section (Figure 6b), which reveals a clear transition from a coarse to a fine microstructure within the first few microns, indicating localized strain accumulation and increased defect density. These defects create a highly active nucleation interface, reinforcing the hypothesis that mechanical pretreatment enhances electrochemical reactivity by introducing structurally favorable sites for ion adsorption, reduction and crystal growth. A similar effect is observed in zinc plating, where a high density of grain boundaries provides abundant nucleation sites, accelerating nucleation and growth rates [38]. Thus, although mechanical processing results in a smoother surface that might intuitively suggest fewer nucleation sites, the introduction of defect-induced activation enhances nucleation density.

Unlike Pu et al. [34], who explored the electrodeposition morphology of Ca via in-situ transmission electron microscopy and reported a current density-dependent transition from island to dendritic growth between 10 to 100 mA cm^−2^, we focused on moderate current densities (0.1, 0.5 and 2.0 mA cm^−2^) to assess their impact on Ca deposit formation at a rotational speed of the stirring bar of 500 rpm. Our results, illustrated by the SEM images in Figure 4c–e, show that the deposits formed within this current density range are far from the aforementioned transition zone. As a result, they primarily consist of coalesced Ca islands. The fraction of coverage was significantly influenced by the current density, increasing from 29% to 55% as the current density increases (Figure 3).

**Figure 7:**
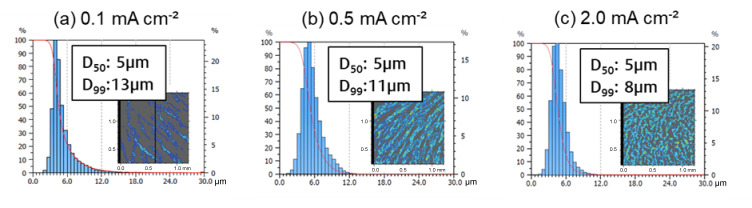
Distribution of Ca islands at varying current densities, measured using confocal microscopy: (**a**) 0.1 mA cm^−^², (**b**) 0.5 mA cm^−^² and (**c**) 2.0 mA cm^−^², each at a charge density of 0.5 mAh cm^−^².

Confocal measurements (Figure 7) confirm these results, by observing a decrease in Ca island height with increasing current density, and vice versa. This observation can be attributed to an increase in nucleation rate and a reduction in island height growth, driven by the enhanced metal ion flux towards the electrode. These results align with the general understanding of the current density dependence. Although the results indicate that increasing the current density can improve substrate coverage, it often leads to undesirable side effects, such as increased electrolyte degradation and the formation of unwanted secondary products. These effects can shorten the effective lifetime of the electrolyte, ultimately impacting the long-term stability of the anode and the overall battery performance. Therefore, we selected a moderate current density of 0.5 mA cm^−^² for the electrodeposition processes, as it provides sufficient surface coverage while maintaining controlled deposition kinetics.

The influence of hydrodynamics on the morphology of electrodeposited Ca was studied at different stirring rates (0 rpm, 250 rpm and 500 rpm) under a current density of 0.5 mA cm^−2^ (Figure 4f–h). Within the conditions tested, the results show that hydrodynamics has a limited effect on the fraction of coverage, which increases from 40% to 50% as the stirring speed is raised from 0 rpm to 500 rpm (Figure 3). However, a significant morphological change is observed when moving from a non-stirred to a moderately stirred electrolyte. Without external stirring (0 rpm), the deposited Ca tends to form a hemispherical structure. According to Guo et al. [39], hemispherical island growth can be usually attributed to mixed diffusion/kinetic control caused by higher overpotentials. These elevated overpotentials restrict the adatom diffusion, thereby inhibiting the formation of well-defined crystal facets and leading to the development of hemispherical islands. In contrast, at moderate stirring speeds (250 rpm) and high stirring speeds (500 rpm), a change towards a coalesced Ca island growth pattern is observed. This can be attributed to the compensation of ion depletion at the electrode interface by decreasing the thickness of the diffusion layer via convection, which lowers the overpotential (for mass transport control) and contributes to the attachment of adatoms to the island periphery, thus contributing to the lateral growth. [39] The findings of Matias et al. [40], who classify Ca(BH_4_)_2_/THF as a ’poor’ electrolyte due to its very low concentration of dissociated Ca^2+^ ions (<2%), provide a plausible explanation for the significant impact and role of hydrodynamics in this context.

Deposits obtained at three defined time intervals (0.25 h, 0.5 h and 2.0 h) were selected to study the growth behavior of Ca islands at a current density of 0.5 mA cm^−2^ over a nominal area of 12.56 mm^2^. These time intervals correspond to charge densities of 0.125, 0.250 and 1.000 mAh cm^−2^, respectively. As illustrated in Figure 4i–k, the plated substrate area expands linearly with increasing charge density. At the early stage of deposition (0.125 mAh cm^−2^), isolated Ca islands are observed, covering approximately 18% of the substrate surface. As the charge density increases to 0.250 mAh cm^−2^, the coverage fraction rises to 42%. After 2.0 h of deposition (1.000 mAh cm^−2^), the coalescence results in approximately 80% coverage of the substrate surface (Figure 4k). This test series clearly illustrates the deposition mechanism from nucleation through island growth to coalescing of these islands. Based on the results from the parameter study discussed above, a Ca anode was electrodeposited under the conditions outlined in Table 2.

**Figure 8:**
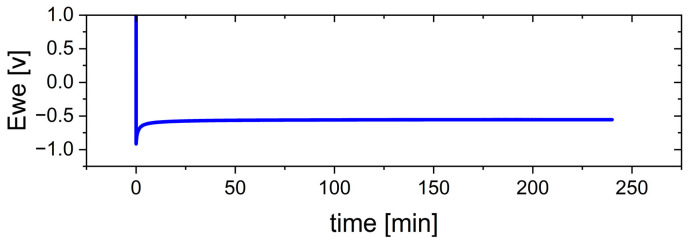
Voltage over time during electrodeposition process of the Ca anode.

Figure 8 illustrates the cell voltage during the electrodeposition process of the Ca anode at a current density of 0.5 mA cm^−2^ for 4.0 h, corresponding to a charge density of 2.0 mAh cm^−2^. A distinct nucleation peak is observed, followed by a stable voltage plateau, which indicates the stable growth of the Ca layer after the initial nucleation phase.

**Figure 9:**
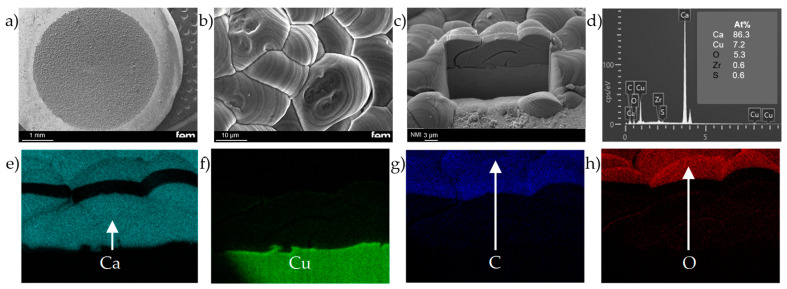
SEM characterization of representative electrodeposited Ca anode to an areal capacity of 2.0 mAh cm^−2^ at 0.5 mA cm^−2^: (**a**) plan-view of the Ca electrodeposit onto Cu substrate; (**b**) enlarged plan-view highlighting the lamellar structure of the Ca deposit; (**c**) cross-sectional FIB-SEM image of the Ca deposit; (**d**) EDX spectrum of the bulk layer, illustrating the elemental composition of electrodeposited Ca anode; and (**e**–**h**) EDX elemental distribution maps for Ca, Cu, C and O, respectively.

The morphology of the electrodeposited Ca anode is shown in Figure 9b. The deposit is approximately 10 µm thick, as confirmed by the FIB cross-section in Figure 9c, and forms a layer of overlapping grains, with small gaps separating individual grains. EDX analysis (Figure 9d) indicates a composition primarily of Ca (86%) with smaller amounts of O (5%) and Cu (7%), consistent with recent findings [41,42]. In comparison, Figure S4 shows a Ca anode made from pressed powder. The bulk material of this anode exhibits an average O-content of 20%, which may limit the performance in battery applications. Based on the measured thickness of the electrodeposited Ca layer, the calculated cathodic current efficiency exceeds 90%. This is in good agreement with previously reported data [28,34,42].

**Figure 10:**
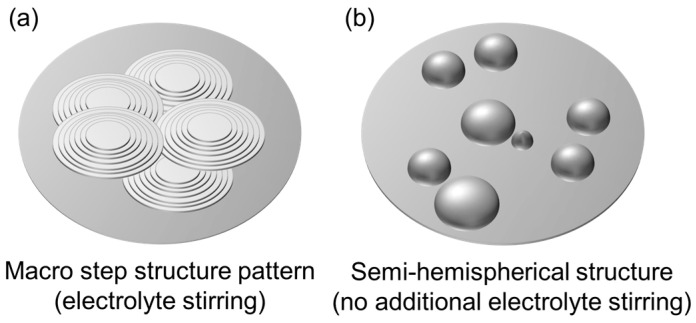
Growth pattern of electrodeposited Ca under (**a**) charge transfer control and (**b**) diffusion control.

The compact deposit demonstrates good adhesion to the Cu substrate and consists of closely packed grains with well-defined grain boundaries. As shown in Figure 9b, each grain is sub-rounded in shape, ranging from 5 to 15 µm in diameter. The outer shape of the grains exhibits a characteristic growth pattern, with the formation of either a lamellar or ring structure, depending on the preferred growth direction. Occasionally, spiral growth patterns were also observed, as indicated in Figure S2. A compact and fine grained layer is typically attributed to 3D growth behavior induced by medium to high overpotential conditions [27]. One possible explanation for these conditions is a limited ion supply, which aligns with recent findings [28]. However, the lamellar pattern observed in our studies is a new observation. It is known that 2D growth behavior can produce so-called macro steps. These patterns can be explained by stepwise growth. According to Winand et al. [43], this may be due to increased inhibition. Nevertheless, as inhibition effects were not specifically examined in this study, a definitive conclusion cannot be drawn without further investigation. The main findings, however, are summarized in a simplified manner in Figure 10. It is important to note that these results require mechanical pretreatment of the substrate. Without this step, the adhesion of the deposit is insufficient, potentially leading to detachment (see Supplementary Figure S3).

## 4. Conclusions

This study systematically investigated the electrodeposition behavior of Ca using a 1.0 M of Ca(BH_4_)_2_/THF electrolyte, demonstrating the potential of electrodeposition as an effective pathway fabricating a thin compact Ca layer for the targeted use as balanced battery anode. Key parameters, including substrate pretreatment, current density, hydrodynamics and charge density, were evaluated to optimize the conditions for producing compact and uniform Ca deposits. Mechanical pretreatment emerged as a critical factor, significantly enhancing nucleation density and coverage compared to chemical pretreatment, enabling the formation of a more uniform and extensive Ca layer. Higher current densities proved advantageous for achieving improved substrate coverage, while hydrodynamics primarily influenced the deposit morphology, transitioning from hemispherical structures to coalesced island growth at higher stirring speeds. Additionally, increasing the charge density led to a nearly linear improvement in substrate coverage, achieving close to 100% coverage with extended deposition times. Our future research will focus on refining the electrodeposition process, incorporating suitable additives and evaluating electrode other electrode substrate materials and interlayers. These advancements are essential for enhancing deposition efficiency, minimizing energy losses from side reactions and validating the practical applicability of the developed anodes.

## Figures and Tables

**Table 1:** Overview of the studied electrodeposition process parameters.

Test Series nr.	Process Parameter	Parameter Value
1	Pretreatment	Chemical, mechanical
2	Current density	0.1, 0.5, 2.0 mA cm^−2^
3	Electrolyte stirring	0, 250, 500 rpm
4	Charge density	0.125, 0.25, 2.0 mAh cm^−2^

**Table 2:** Electrodeposition parameters for the fabrication of a compact Ca anode with a layer thickness of approx. 10 µm.

Electrodeposition Parameter	Parameter Value
Pretreatment	Mechanical
Current density	0.5 mA cm^−2^
Electrolyte stirring	500 rpm
Charge density	2.0 mAh cm^−2^
Plating time	4.0 h

## Data Availability

The original contributions presented in this study are included in the article/Supplementary Material. Further inquiries can be directed to the corresponding author.

## Supplementary Materials

The following supporting information can be downloaded at: https://www.mdpi.com/article/10.3390/nano15060454/s1. Figure S1: The Trainable Weka Segmentation machine learning software is employed to extract quantitative data from SEM images of Ca deposits. The process involves training the algorithm to recognize and segment specific features within the images, such as grain boundaries and other morphological characteristics. The segmented data is then analyzed to obtain precise measurements and statistical information about the Ca deposits, including the fraction of substrate coverage, thereby providing a deeper understanding of their structural properties. Figure S2: SEM characterization of a representative electrodeposited Ca anode to an areal capacity of 3.0 mAh cm^−2^ at 0.5 mA cm^−2^ shows a spiral growth pattern prominently located slightly to the left of the the centre of the image. Figure S3: SEM characterisation of the electrodeposited Ca layer on a chemically pretreated Cu substrate shows weak adhesion between the Ca layer and the substrate, resulting in the formation of cracks and delaminations. The white region observed near the center right of the Ca deposit is indicative of localized electron charging, suggesting inadequate contact between the Ca layer and the underlying substrate. Figure S4: SEM characterization of representative powder based Ca anode (a) Plane view of the Ca anodee (b) Enlarged plane view highlighting the detailed structure of the Ca anode, (c) Cross sectional SEM image showing the bulk structure of the Ca anode (d) EDX spectrum of the bulk layer, illustrating the elemental composition of the Ca anode. Table S1: Detailed overview of the electrodeposition experiments.
