# Supplementary material for: Towards Thin Calcium Metal Anodes—An Essential Component for High-Energy-Density Calcium Batteries
Source: Nanomaterials (Basel). 2025 Mar 17;15(6):454. doi: 10.3390/nano15060454 (PMC11944523; doi:10.3390/nano15060454)
Supplement: Supplementary file 1 [file nanomaterials-15-00454-s001.zip › nanomaterials-3474898-supplementary.pdf]

# Ultrathin Calcium Metal Anode – An Essential Component for High-Energy-Density Calcium Batteries

Christoph Kiesl[1,4*], Reinhard Böck[1], Holger Kaßner[1], Joachim Häcker[2], Marco Kögel[3], Timo Sörgel[4] and Şeniz Sörgel[1*]

[1]Department of Electrochemical Energy Systems, fem Research Institute; Katharinenstrasse 17, 73525 Schwäbisch Gmünd, Germany

[2]Institute of Engineering Thermodynamics, German Aerospace Center (DLR), Pfaffenwaldring 38-40, 70569 Stuttgart, Germany

[3]Natural and Medical Sciences Institute at the University of Tübingen (NMI), Markwiesenstraße 55 Reutlingen, Germany

[4]Center for Electrochemical Surface Technology (ZEO), Aalen University of Applied Sciences, Beethovenstr. 1, 73430 Aalen, Germany

*Correspondence: kiesl@fem-online.de

## Electronic Supplementary Information

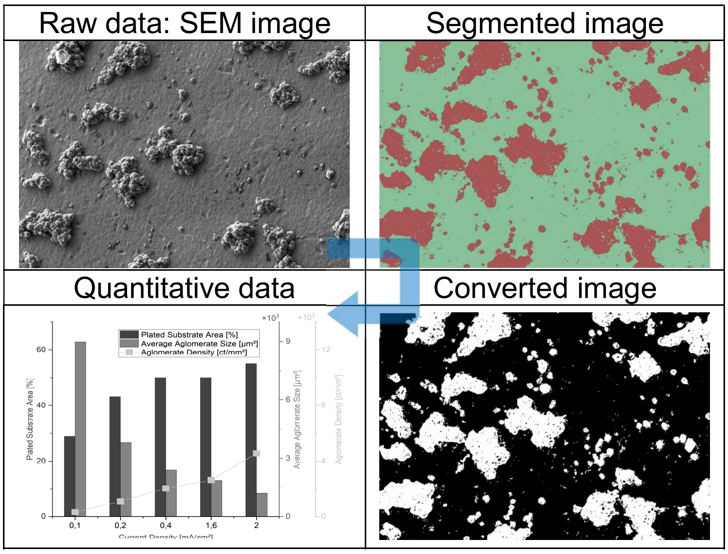


*Figure S1: The Trainable Weka Segmentation machine learning software is employed to extract quantitative data from SEM images of Ca deposits. The process involves training the algorithm to recognize and segment specific features within the images, such as grain boundaries and other morphological characteristics. The segmented data is then analyzed to obtain precise measurements and statistical information about the Ca deposits, including the fraction of substrate coverage, thereby providing a deeper understanding of their structural properties.*

*Table S1: Detailed overview of the electrodeposition experiments.*

| Nr. | Pre-treatment | Current density [mA·cm⁻²] | Stirring rate [rpm] | Plating time [h] | Charge density [mAh·cm⁻²] |
|---|---|---|---|---|---|
| 1.1 | Mechanical | 0.5 | 500 | 0.5 | 0.25 |
| 1.2 | Chemical | 0.5 | 500 | 0.5 | 0.25 |
| 2.1 | Mechanical | 0.1 | 500 | 2.5 | 0.25 |
| 2.2 | Mechanical | 0.5 | 500 | 0.5 | 0.25 |
| 2.3 | Mechanical | 2.0 | 500 | 0.125 | 0.25 |
| 3.1 | Mechanical | 0.5 | 0 | 0.5 | 0.25 |
| 3.2 | Mechanical | 0.5 | 250 | 0.5 | 0.25 |
| 3.3 | Mechanical | 0.5 | 500 | 0.5 | 0.25 |
| 4.1 | Mechanical | 0.5 | 500 | 0.25 | 0.125 |
| 4.2 | Mechanical | 0.5 | 500 | 0.5 | 0.25 |
| 4.3 | Mechanical | 0.5 | 500 | 2.0 | 2.0 |

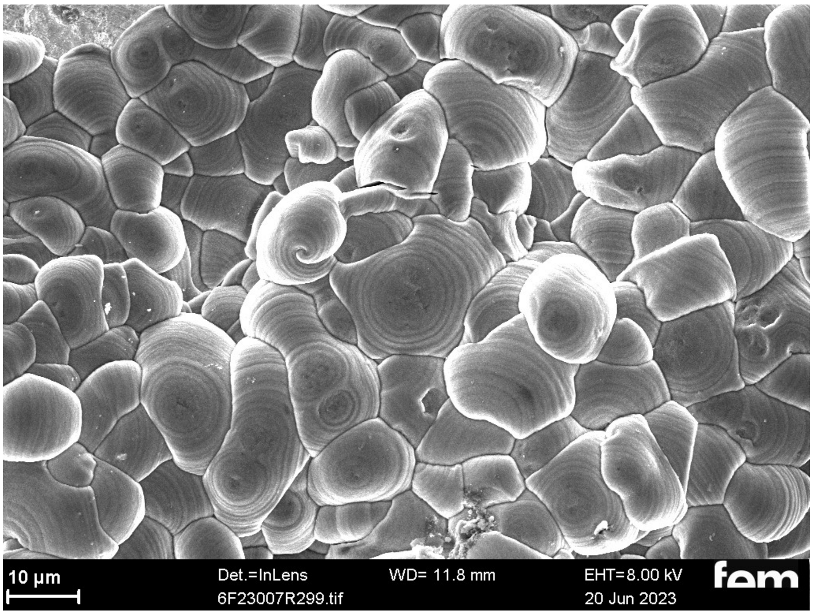


*Figure S2: SEM characterization of a representative electrodeposited Ca anode to an areal capacity of 3.0 mAh cm$^{-2}$ at 0.5 mA cm$^{-2}$ shows a spiral growth pattern prominently located slightly to the left of the the centre of the image.*

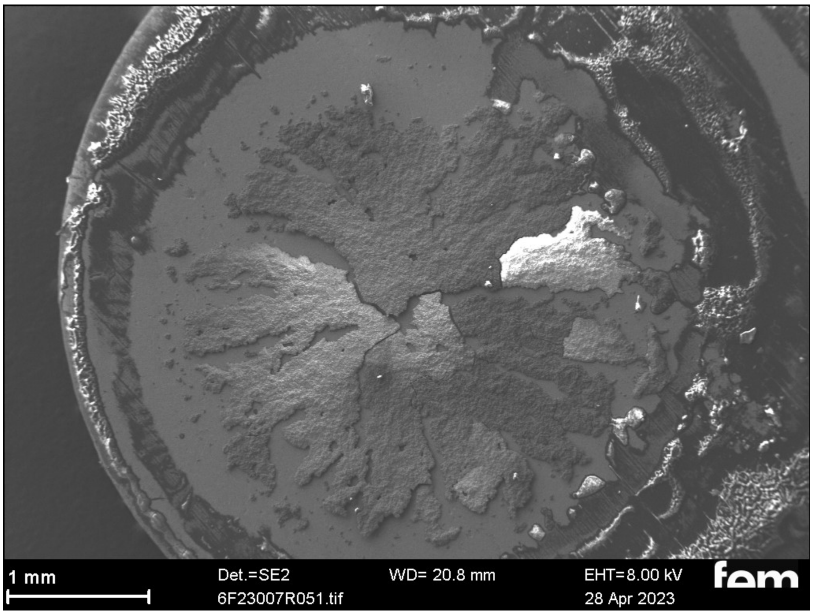


*Figure S3: SEM characterisation of the electrodeposited Ca layer on a chemically pretreated Cu substrate shows weak adhesion between the Ca layer and the substrate, resulting in the formation of cracks and delaminations. The white region observed near the center right of the Ca deposit is indicative of localized electron charging, suggesting inadequate contact between the Ca layer and the underlying substrate.*

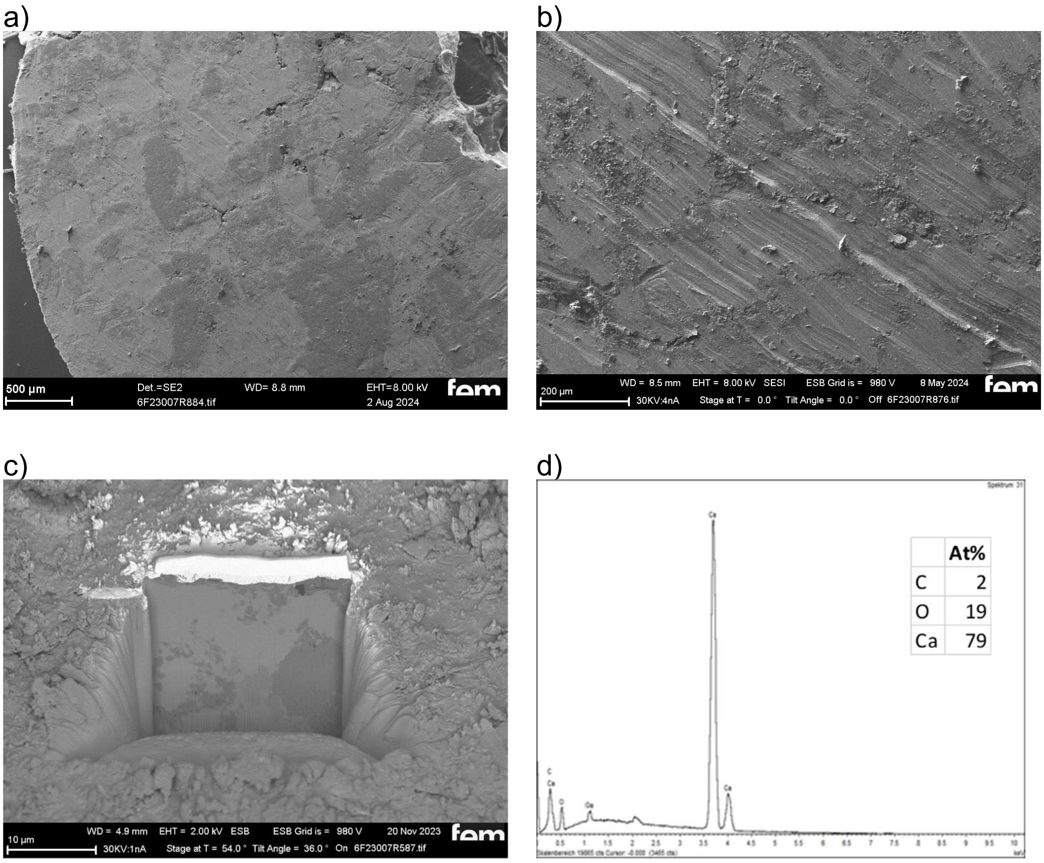


*Figure S4: SEM characterization of representative powder based Ca anode (a) Plane view of the Ca anodee (b) Enlarged plane view highlighting the detailed structure of the Ca anode, (c) Cross sectional SEM image showing the bulk structure of the Ca anode (d) EDX spectrum of the bulk layer, illustrating the elemental composition of the Ca anode.*
